# Time Goes Back—Time Perspective in Polish Men with Compulsive Sexual Behavior Disorder and Risky Sexual Behavior

**DOI:** 10.3390/ijerph20064954

**Published:** 2023-03-11

**Authors:** Julia Wyszomirska, Monika Bąk-Sosnowska

**Affiliations:** 1 Department of Psychology, Faculty of Health Sciences in Katowice, Medical University of Silesia in Katowice, 40-752 Katowice, Poland; 2 Center for Psychosomatics and Preventive Healthcare, WSB University in Dąbrowa Górnicza, 41-300 Dąbrowa Górnicza, Poland

**Keywords:** risky sexual behavior, sexual addiction, compulsive sexual behavior disorders, time perspective, behavioral addictions

## Abstract

Many relationships between time perspective and a propensity to engage in risky behaviors or developing addictions have been demonstrated. The aim of our study was to determine the differences in intensity of individual time perspectives in people with compulsive sexual behavior disorder (CSBD) and risky sexual behavior (RSB). The analysis includes 425 men: 98 CSBD (age M = 37.99 years), 63 RSB (age M = 35.70 years), 264 without CSBD and RSB features constituting the control group (age M = 35.08 years). We used the Zimbardo Time Perspective Inventory, the Sexual Addiction Screening Test—Revised, the Risky Sexual Behavior Scale and a self-constructed survey. The comparative analysis showed a higher intensity of past-negative (*p* = 0.040), a lower of past-positive (*p* < 0.001) and a present-fatalistic (*p* = 0.040) outlook in the CSBD group compared to the control group. Compared to the participants with RSB, the CSBD group was characterized by a higher intensity of past-negative (*p* = 0.010), a lower of past-positive (*p* = 0.004) and a present-hedonistic perspective (*p* = 0.014). The RSB group also achieved higher results from the present-hedonistic perspective (*p* = 0.046) compared to the control group. The CSBD patients indicate a stronger tendency to focus on negative past compared to non-CSBD men, both taking and not taking RSB. The time perspective profiles of RSB men are similar to those who do not engage in RSB. The distinguishing feature of men with RSB without CSBD is a greater ability to enjoy current experiences.

## 1. Introduction

Risky Sexual Behavior (RSB) and Compulsive Sexual Behavior Disorder (CSBD) have both common and distinct characteristics. In general, RSB often affects people with CSBD; however, not all people engaging in RSB will develop CSBD. From the point of the need to understand the etiology, maintaining the above-mentioned behaviors, and looking for treatment options, it is important to understand whether the similarity at the behavioral level (CSBD and RSB) is also related to similar etiological factors and psychological characteristics. Or is there something specific for each of the groups, what makes that some people, even those engaging in numerous RSB, will not show the features of addiction or compulsivity, while others will develop these features? One of the psychological features that turned out to be significant in disorders due to substance use and addictive behaviors development or the use of risky behaviors is the time perspective (TP).

In general, RSB can be defined as sexual behaviors that increase the likelihood of negative health outcomes (e.g., sexually transmitted infections—STIs; unintended pregnancy; oncological diseases, including head and neck, cervical, vulvar, vaginal, prostate and oral cancer) and psychosocial effects (e.g., family conflicts, broken relationships, legal disputes, financial problems) [1,2,3,4,5,6,7]. The specific, most frequently mentioned and studied forms of RSB include lack of condom use and contraception, having a large number of sexual partners in a lifetime, non-discriminating sexual partner recruiting patterns, participating in concurrent sex partnerships, maintaining several sexual relationships at the same time and having sex after heavy consumption of alcohol or other psychoactive substances [2,8]. Thus, RSB has become one of the leading causes of the global health burden [9].

So far, the following risk factors for RSB have been identified: younger age (adolescents and young adults) [10,11]; male gender [12]; mental disorders [13], especially borderline personality [14] and substance use disorders [15], and even substance use itself [12,16]; being a victim or witness of physical abuse [16,17]; childhood sexual abuse experience [18]; and genetic factors [3,19]. 

With increasing evidence suggesting that loss of control over sexual behavior is a significant clinical problem that, if untreated, can lead to serious negative outcomes, the International Statistical Classification of Diseases and Related Health Problems (ICD-11) has introduced a category of CSBD. “It is characterized by a persistent pattern of failure to control intense, repetitive sexual impulses or urges, resulting in repetitive sexual behavior over an extended period (e.g., six months or more) that causes marked distress or impairment in personal, family, social, educational, occupational or other important areas of functioning” [20]. The pattern may include: (1) repetitive sexual activities becoming a central focus of the person’s life to the point of neglecting health and personal care or other interests, activities and responsibilities; (2) numerous unsuccessful efforts to significantly reduce repetitive sexual behavior; and (3) continued repetitive sexual behavior despite adverse consequences or (4) deriving little or no satisfaction from it. Distress resulting solely from moral judgments and disapproval of sexual impulses, urges, or behaviors are not sufficient to meet a diagnosis of CBSD, and paraphilic disorder is an exclusion factor [21].

The etiology of CSBD is not well understood. The main identified reasons include the increasingly easy access to pornographic materials and cybersex via the Internet [22,23] or social changes that led to the discontinuation of treating sexuality as taboo in Western culture [24]. CSBD often co-occurs with other mental disorders, i.a., mood and anxiety disorders, substance use disorders [25,26,27] and experience of sexual abuse in childhood [28,29]. There is a lack of data on CSBD prevalence in the general population. Ranges are estimated widely from 3 to 6% [30], wherein the problem affects men much more often than women [31,32]. Solid data on differences in this area are still insufficient [20,33]. In addition, higher rates of CSBD are found in people with substance use disorders [20]. People with CSBD often present intensive RSB [34,35,36,37]. However, their presence is neither sufficient nor even necessary for the CSBD diagnosis. Their meaning is expressed in the context of connection with symptoms typical of addiction or compulsivity. Both in the ICD-11 and in proposals for hypersexuality disorders recommended for the DSM-5, as well as in Carnes’ sexual addiction classification, which is popular among clinicians, they can be found in diagnostic criteria relating to the negative consequences of RSB in various areas of functioning [21,30,38].

Temporal orientation is a psychological process that makes it possible to maintain a sense of continuity of one’s own experiences, which is the basis for creating and maintaining the constancy of a sense of identity, understanding of the surrounding world, as well as the ability to plan and realize goals. Accordingly, the reality assigned to the past, present and future is evaluated subjectively. This personal attitude toward time is referred to as the dominant TP [39,40]. Perhaps the most popular current approach to studying individual differences in psychological time is Zimbardo and Boyd’s (1999) Time Perspective Theory (TPT) [39]. The authors define TP as “the often nonconscious process whereby the continual flows of personal and social experiences are assigned to temporal categories, or time frames, that help to give order, coherence, and meaning to those events” [39] (p. 1271). TP is thus a continuous cognitive process involving the allocation of attentional resources between different temporal horizons. On the other hand, individuals can build stable, dispositional tendencies to use or overuse a particular temporal orientation, developing TP as a trait. According to TPT, a person’s personal attitude toward time is determined by a number of variables, which are mainly non-biological, including culture, religion, membership of a particular social group, economic conditions, values dominant in the course of upbringing, education and personal experience [39,40]. Research on TP, and its factor structure, has led to the identification of five attitudes towards time.

Past-negative: The tendency to constantly mentally revisit and relive negative past experiences, avoid change and carefully retain memories of one’s failures. Individuals characterized by a dominant past-negative perception have few close friends and a tendency to be depressive, experiencing frequent anxiety, aggression and low self-esteem [39,40,41,42]. This orientation is associated with mental disorders [41], substance use disorders [43] and low life satisfaction [44].

Past-positive: The tendency to frequently return to positive experiences and pleasant memories. These returns are sometimes sentimental, nostalgic, but tinged with positive emotions and are used to achieve well-being in the present. This attitude is associated with good mental health, self-esteem [39,40,41,42], as well as life satisfaction [44], a sense of happiness [45] and health-promoting behaviors [46].

Present-hedonistic: An ease of finding, enjoying and appreciating current events and nice moments; associated with higher levels of life satisfaction and well-being in people with a present-hedonistic mindset [45,47], but also with intentionally seeking intense stimulation, pleasure, activity that guarantees immediate gratification, without reckoning with distant consequences [39,40,41,42]. This attitude can promote risky behavior and addiction [43,48,49,50].

Present-fatalistic: A poor sense of influence over one’s life and a belief that current situations and experiences are dependent on unidentified external factors, such as “luck,” “fate,”, etc. [39,40]. Higher levels characterize people with a tendency for mental disorders, substance use disorders [39,40,41,42] and a crisis of homelessness [51].

Future: Constantly striving to plan and achieve goals and believe in achieving them [39,40]; associated with less problematic behavior [52], although sometimes at the expense of losing spontaneity and the ability to enjoy current events [40].

Each perspective determines a different value hierarchy, emotional attitudes toward self and people, sensitivity to a specific form of gratification and response with behaviors in the face of changing life situations. The extent to which an individual’s temporal orientation will work to a particular person’s advantage depends on both one’s characteristic relatively stable profile, and the flexibility and appropriateness of using a particular TP in specific situations [40,53,54].

The term used to describe the optimal, most adaptive temporal orientation is balanced time perspective (BTP). This is defined as the mental ability to effectively switch between temporal perspectives depending on the nature, task characteristics, situational conditions and personal resources [40]. The concept was refined by the introduction of the optimal BTP profile, characterized by low levels of present-fatalistic and past-negative, moderately high present-hedonistic and future, and high scores on the past-positive scale [40]. Subsequent studies confirmed the relevance of the concept, determining its relevance to health, well-being [45,52,53,54] and deviations from it as a factor associated with poorer mental and physical functioning, quality of life, poorer personal achievement, etc. [54,55]. 

Both the TPT itself and the results of research conducted to date in the context of risk behavior [48,50,56,57,58], substance use disorders and addictive behaviors [56,59,60,61,62,63,64,65,66,67,68,69,70,71,72,73,74], mood, psychological states or features and personality traits [41,47,51,52,63,75,76,77,78,79,80,81,82,83] allow formulation of the conjecture that TP plays an important role in RSB and CSBD. To date, several studies have appeared looking at its direct or indirect links to RSB [48,84,85,86,87]. Common neuroanatomical correlates are being considered as a potential explanation for the co-occurrence of present TP and risky behaviors [88]. A future perspective minimizes the tendency to engage in RSB [48,84]. Future-oriented individuals, even in spite of perceived desire, will be more likely to delay the immediate gratification that risky sexual contact provides. They realize that indulging in this sexual temptation may interfere with their long-term goals [89]. Conversely, present TP (i.e., driven by immediate rewards or law sense of efficiency) exacerbates the tendency to engage in RSB [48,86]. 

An analysis of past studies indicates similar shortcomings as those observed for other risk behaviors: focusing mainly on adolescents and young adults and behaviors that increase risk of STIs or unintended pregnancy. This is justified from the point of view of public health and knowledge of developmental psychology, but it does not allow for extrapolation of the aforementioned results to people at other stages of development and using other RSB that involve, for example, negative psychological, social or financial consequences (infidelity, excessive use of pornography). There is still a gap in this area.

Disorder due to substance use and engaging in risky behaviors were the first and still remain important areas of research in relation to TPT. Over the years, addictive behaviors have been added to the ranks of increasingly addressed problems. The importance of TP has been demonstrated in Internet and social media addiction [67,68,70,73,90,91,92], gambling disorder [64,65,66,93,94,95], compulsive buying [62] or food addiction [74]. However, to date, no studies have been conducted or published on the relationship between TP and CSBD (based on database searches: APA PsycInfo, Academic Search Ultimate, Medline, SocINDEX with Full Text, Health Source: Nursing/Academic Edition, OpenDissertations MasterFILE Premier, Business Source Ultimate, The Belt and Road Initiative Reference Source, eBook Academic Collection, eBook Collection, APA PsycArticles, ERIC, searching the databases with the various terms used to describe CSBD prior to its entry into ICD-11).

Studies including people with clinical diagnosis are very rare (see [66]) and researchers in their own projects often recognize this lack and call for the inclusion of such groups, e.g., Kim et al. [68]. As with risky behavior, most researchers focus on people in adolescence or young adults. As is known from other studies, TP changes with age, varying at different stages of development [96,97]. Additionally, age modifies the effect of temporal perspective on other psychological variables [98]. Given the above factors, the main objective of our study was to analyze the relationship between TP characteristics and RSB and CSBD. The following research hypotheses were put forward: There are differences in the severity of particular TP between individuals with CSBD and the control (C) group. There are differences in the severity of particular TP between individuals with CSBD and those undertaking RSB. The severity of particular TP in the group with RSB is more similar to the results of the C group than individuals with CSBD. 

## 2. Materials and Methods

### 2.1. Participants

**Figure 1:**
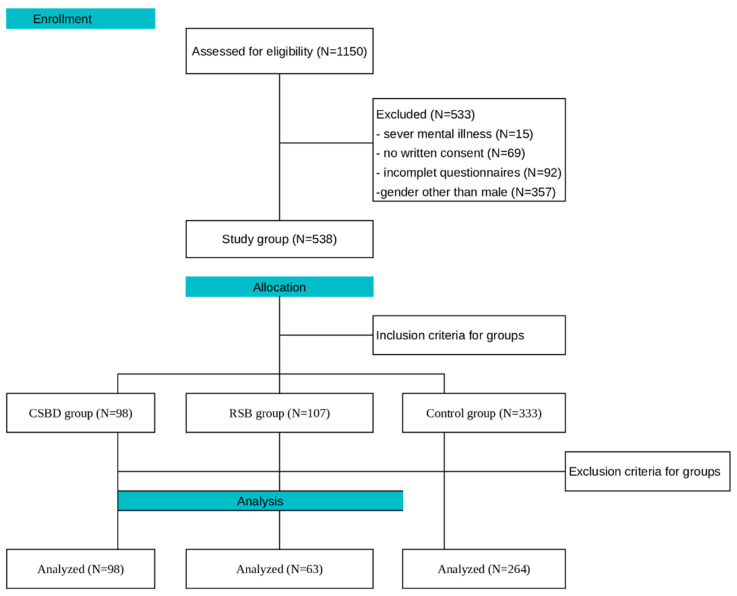
Stages of classification into study groups based on inclusion and exclusion criteria.

In order to compare differences in TP in men with CSBD, undertaking RSB and individuals without CSBD and RSB, the study included the three aforementioned groups. Common inclusion criteria for all subjects were an age of 18 or older and consent to participate in the study. Exclusion criteria included: confirmation of a diagnosis of serious mental illness (e.g., schizophrenia), dementia or mild cognitive impairment, dopaminergic therapy, failure to confirm written consent or withdrawal of consent to participate in the study, incomplete completion of the study questionnaire, preventing analysis. At this stage of the study, those declaring a gender other than male were also excluded. This was due to the fact that CSBD is more often diagnosed in men than in women, and initial inquiry indicated that almost exclusively men were attending therapies. However, we are currently collecting data on women, which requires more time and expanded collaboration with more centers. Table 1 presents group-specific inclusion and exclusion criteria and the diagram in Figure 1 shows the exclusion steps, along with their criteria. 

### 2.2. Methods

We used the diagnostic survey method. All participants were given the same research questionnaire, consisting of screening and standardized psychological tests and a self-report survey questionnaire. The research questionnaire included: 

The Zimbardo Time Perspective Inventory (ZTPI) [39], in the Polish adaptation of Kozak and Mażewski [99], was used to assess individual TP. It consists of 56 items which require the participant to respond on a 5-point Likert scale from “Completely disagree” to “Completely agree.” The items form 5 scales: Past-positive, Past-negative, Present-hedonistic, Present-fatalistic, and Future. The theoretical basis of the tool is TPT [39]. Scores on each scale were additionally used to determine the deviation from the balanced time perspective (DBTP) [77].

The Sexual Addiction Screening Test-Revised (SAST-R) by Carnes et al. [100] in the Polish adaptation of w Gola et al. [101]. The questionnaire was used to assess the level of loss of control over sexual behaviors. It contains 20 items to which the respondent is asked to respond by giving a “Yes” or “No” answer. The psychometric parameters of the original tool are comparable to the Polish version [100,101]. In the Polish adaptation, the optimal cutoff point is 5 points, for which the sensitivity is 99.1% and specificity is 78.3%. A score after the cutoff point can be interpreted with high probability as the absence of CSBD. On the other hand, classifying patients with CSBD on the basis of a score of 5 points and above is not so clear-cut. The above limitations of the tool were taken into account in the planned study. It was not used to qualify for the clinical group, but to exclude individuals from the control and RSB groups.

Risky Sexual Behavior scale (RSBs) by Verweij et al. [3,19], self-translated with proofreading, was used to assess the severity of RSB. It includes a checklist of 8 high-risk behaviors, such as failure to use condoms or other birth control methods, participating in concurrent sexual relationships, non-discriminating sexual partner recruitment, having sex with more than one person in a 24 h period, engaging in sexual behavior after heavy alcohol consumption and ever having had a disease due to STIs. These behaviors have been identified as increasing the risk of STIs and unintended pregnancy and correlate to forming a pattern of risky behaviors. Participants are asked to tick all behaviors they have ever exhibited. A total RSBs is calculated by summing the checked behaviors. In addition, the number of sexual partners in a lifetime is taken into account. Those declaring three to ten partners receive an additional point, while those who state more than ten receive two points. Hence, RSBs scores range from 0 to 10 points [3,19]. 

To verify if the measurement with the tools used had an acceptable level of reliability, Cronbach’s α coefficients were calculated. As shown in Table 2. the reliability for all scales was satisfactory (α > 0.7). 

The self-survey questionnaire consisted of 14 items, including open-ended and closed-ended questions, allowing the collection of information on:

- Demographic data, including: age, gender, education, occupational activity, relationship; psychosexual orientation;
- Clinical data, including: diagnosed disorder due to substance use or addictive behaviors, other mental disorders; somatic diseases, medications taken;
- Dominant sexual behaviors, i.e., sexual contact, use of use of pornography, other;
- Questions only for participants with CSBD, i.e., approximate date of diagnosis, the number of therapies undertaken to date.

### 2.3. Study Organization

A pilot study was conducted in January and February 2019, with the aim of verifying the correct selection of research tools and the recruitment and organization of the study itself. In total, 19 men between the ages of 23 and 52 (M = 37.5; SD = 9.6) participated, 15 of whom had a diagnosis of CSBD. Appropriate data were collected from December 2019 to January 2021, with periods appearing when data collection was significantly hampered due to COVID-19 pandemic restrictions. Subjects in the clinical group were patients of all centers offering CSBD treatment in the Silesian and Lower Silesian provinces—addiction treatment facilities in Poland. Individuals for the RSB and C group were recruited using the snowball method, starting with friends, families and acquaintances of students at the Medical University of Silesia and purposively at the HIV counseling and diagnostic center and infectious disease clinic in Silesia. 

The survey was anonymous, confidential and voluntary. There was no payment for participation. Informed consent was obtained from all subjects involved in the study. At the beginning of the survey, information about the purpose and procedure of the study and the possibility of direct contact with the investigator was provided. In order to increase participants’ comfort, the questionnaire was completed individually and there was no time limit. At the end, respondents placed the questionnaire in an envelope and handed the sealed envelope to the person conducting the study directly or indirectly (through, for example, a doctor, therapist or academician). Provision was made for subjects to opt out of participating in the study by not returning or destroying the completed questionnaire.

### 2.4. Statistics

To verify the research hypotheses, we carried out statistical analysis using the IBM SPSS Statistics 25 software. Using this tool, we performed frequency analysis, basic descriptive statistics together with the Kolmogorov–Smirnov test to examine if the variables were normally distributed. Due to the unequal nature of the study groups, the Kruskal–Wallis test was performed to assess differences in the intensity of individual TP. If the result was statistically significant, it meant that at least one group was different from the other. Then, post hoc analyses were performed using Dunn–Sidak tests to check which group differed from each other. The standard significance level *p* < 0.05 was adopted. 

DBTP was calculated based on comparisons of the averages obtained in our own research for the individual scales of the ZTPI with the optimal scores for BTP, taken from Zimbardo and Boyd’s cross-cultural database [40]. It was based on the formula proposed by Stolarski, Bitner and Zimbardo [77]:

$$DBTP=\sqrt[{}]{{\left(oPn-ePn\right)}^{2}+{\left(oPp-ePp\right)}^{2}+{\left(oPf-ePf\right)}^{2}+{\left(oPh-ePh\right)}^{2}+{\left(oF-eF\right)}^{2}}$$

*DBTP*—Deviation from the balanced time perspective; *Pn*—past-negative; *Pp*—past-positive; *Pf*—present-fatalistic; *Ph*—present-hedonistic; *F*—future; *e*—empirical score; *o*—optimal score.

## 3. Results

### 3.1. Study Groups Characteristics

Finally, the results of 425 men (age M = 34.80; SD = 10.67; Median = 33; IQR = 19) who could be assigned to one of the groups based on the constellation of three criteria (confirmation of CSBD diagnosis, SAST-R score and RSBs score) were included in the analyses. Men with higher education (N = 219; 51.5%) and who were economically active (N = 382; 89.8%) prevailed. The majority characterized themselves as heterosexual (N = 404; 95.0%) and currently in stable partnerships/marriages (N = 331; 77.8%). The group of those who manifested RSB included 63 individuals aged 20 to 63 (M = 35.70; SD = 10.57; Median = 33; IQR = 16), and the C group included 264 men aged 20 to 64 (M = 35.08; SD = 11.26; Median = 32; IQR = 20). The CSBD group was made up of 98 men aged 20 to 61 (M = 37.99; SD = 9.08; Median = 39,5; IQR = 13). A one-way analysis of variance was performed in a between-group design, the result of which was found not to be statistically significant, F(2, 422) = 2.65; *p* = 0.072, so it was assumed that the groups were not significantly different in terms of age. It was decided that further analyses would be performed on the entire sample, without aiming for equality of groups, which would involve a significant reduction in the size of the group and, to a lesser extent, of the CSBD group. Demographic and clinical characteristics of the participants are presented in the following two Table 3 and Table 4.

Men with CSBD were slightly more likely to have confirmed diagnoses of mental disorders and somatic diseases. Additionally, 25.5% of the subjects (N = 25) in the CSBD group confirmed taking at least one medication on a regular basis. None of the respondents confirmed taking dopaminergic medications. A score above the cutoff point on the SAST-R scale identified 85 men (96.9%) with the specialist-confirmed diagnosis of CSBD. Overall, 43 (43.9%) of the men in the CSBD group identified sexual intercourse as their predominant addictive behavior, while 42 (42.9%) identified sexual intercourse and pornography use as most prevalent, and 11 (11.2%) used pornography most often. Two (2%) men indicated practicing “other sexual behaviors” in addition to sexual intercourse and pornography use, but did not specify.

### 3.2. A Comparison of the Intensity of Each Time Perspective in the Study Groups

The results of Kruskal–Wallis tests (Table 5) confirmed four statistically significant results, in terms of the level of: past-negative, present-hedonistic, past-positive and present-fatalistic TP. In addition, DBTP was compared, which also proved to be significantly different in the samples. Therefore, a post hoc analysis was performed using Dunn–Sidak tests. Only in terms of future scale scores were the differences between groups not significant.

CSBD group scores differed significantly from those of both the RSB and C groups, on scales measuring attitudes toward the past. Individuals in the clinical group had higher levels of past-negative TP than those in the RSB (*p* = 0.010) and C group (*p* = 0.040).

In contrast, an inverse relationship was noted with regard to the past-positive TP. Namely, men with CSBD scored lower on the past-positive scale than those who undertake RSB (*p* = 0.004) and those from the C group (*p* < 0.001). At the same time, the results of the latter two groups were not significantly different on either the past-negative (*p* = 0.575) or the past-positive scale (*p* = 1).

With regard to the level of present-fatalistic TP, significantly statistically lower scores were recorded in men with CSBD compared to those in the C group (*p* = 0.040). The differences between the RSB and C group (*p* = 1) and those with CSBD (*p* = 0.068) were not statistically significant. 

The level of present-hedonistic TP was highest in the group of RSB individuals and was significantly different from the results of men with CSBD (*p* = 0.014) and the C group (*p* = 0.046). In contrast, the scores of the two groups were not statistically significantly different (*p* = 1). 

The DBTP value was statistically significantly higher in the CSBD group compared to the C group (*p* = 0.022), but not when compared to the results in the RSB group (*p* = 0.107). There were also no confirmed differences in this regard between subjects in the RSB and C group (*p* = 1).

## 4. Discussion

The purpose of our study was to identify TP in men with CSBD and those undertaking RSB. The obtained results proved that there are differences between the study groups, which is largely in line with the assumptions of the TPT [40] and the results of studies relating to the role of the aforementioned constructs in the development of disorders, including addictive behaviors or engaging in risky behaviors. Traits that appear to be particularly associated with CSBD are both past TP. These traits significantly differentiate individuals with CSBD from both the C and RSB groups. In contrast, a trait that distinguishes RSB individuals from the other studied groups is a higher intensity of hedonistic attitudes toward the present.

From our investigations, it appears that this is the first study on TP in CSBD. Therefore, due to the lack of results from other studies, the discussion refers in more detail to the results of studies in the area of addictive behaviors.

### 4.1. Past-Negative

According to the results of the study, men with CSBD are characterized by a tendency to revisit unpleasant, traumatic memories, failures and difficulties, but also have a tendency to interpret current events, challenges and expectations for the future in the context of negative experiences. This is consistent with results obtained by other researchers, demonstrating the importance of a past-negative in substance abuse and the severity of other psychopathological symptoms [39,40,41,42,82]. 

The high intensity of the past-negative TP can rightly evoke automatic associations with depressive disorders. It is blandly inscribed in the characteristics of cognitive functioning of people with depression. They are noted to have a tendency to recall unpleasant memories more often, along with a tendency to create excessive generalizations of autobiographical memory (poorly recalled details), which translates into the formation of extreme, unquestioning beliefs and the attribution of special responsibility for past failures to themselves [102,103]. Indeed, the research results confirm positive correlations between the severity of depressive symptoms, as well as anxiety and past-negative TP in the general population [40,47,82,104] and clinical groups [83]. Thus, it can be concluded that, in this respect, men with CSBD are similar to people with depressive disorders. However, much of the past-negative TP is also revealed in more specific (and, at the same time, closer to CSBD) clinical problems. 

With regard to disorders due to addictive behaviors, similar results were obtained in Unger et al.’s [62] study. It showed that a higher level of past-negative TP was a predictor of compulsive buying in three groups of students from different cultural backgrounds: Germany (N = 314; age M = 24.3 years), Ukraine (N = 297; age M = 24.0 years) and China (N = 305; age M = 19.7 years). Furthermore, compared to other associations between compulsive buying symptoms and temporal orientation, this relationship was universal, present in each of the study groups. Similarly, a study conducted among 756 FB users (age M= 21.38 years) in the Polish population, which separately assessed the severity of FB and Internet addiction symptoms, also confirmed the role of a past-negative focus in the development of both Internet and FB use disorders [67]. Additionally, a study conducted by Miceli et al. [92] involving 186 social media users (age M = 22.54 years) confirmed that high intensity of past-negative TP increases the risk of social-media use disorder and is associated with all symptoms of addiction, such as excessive preoccupation and time commitment, mood regulation and impairment in social functioning. Not all studies show such direct links. For example, in a study of 233 individuals (age M = 21.4 years), past-negative TP increased the risk of FB use disorder, but only when it co-occurred with high levels of neuroticism [90]. A group more similar in age to the individuals in our study was the 149 Internet users (age, M = 31.40 years) in Chittaro and Vianello’s study [70]. Caplan’s Generalized Problematic Internet Use Scale 2 (GPIUS2) was used to assess the severity of problematic Internet use. The results confirmed the predictive importance of a past-negative TP in the severity of Internet use disorder symptoms (explaining 9% of the variation in GPIUS2 scores). In addition, a higher level of past-negative TP was associated with a stronger tendency to alleviate unpleasant feelings with Internet use (Mood Regulation subscale score) and a preference for online interpersonal contact (Preference for Online Social Interaction subscale score), rated as a safer and more effective means of communication than traditional face-to-face interpersonal contact. Our results are also similar to those noted in the study that determined the relationship between TP and symptoms of food addiction. It included an academic community (N = 949 subjects, age M = 21.8 years) and was conducted during the COVID-19 pandemic restrictions in Russia. Finally, past-negative TP was identified as the best predictor, among time perspectives, of food addiction [74].

The only study that included a clinical group, making it particularly valuable for comparing our own results, is that of Hodgins et al. [66], in which patients with a diagnosis of gambling disorder (N = 20; age M = 42.3 years) were characterized by higher levels of past-negative TP compared to social gamblers (N = 22; age M = 34.9 years), but lower levels in relation to patients with mental disorders (N = 20; age M = 39.5 years) [66].

In contrast with the studies discussed above, our study included only men in the developmental stage of middle adulthood. In addition, CSBD was not operationalized based solely on the questionnaire score, but on a diagnosis made by a specialist. Despite such important differences, the results are comparable, indicating the broad reach of the past-negative TP in the development of disorders, especially behavioral addictions, against the background of the available literature—the first time it has been showed in CSBD. 

### 4.2. Past-Positive

The results suggest that past-positive TP seems to be of particular importance in CSBD. The level of this perspective is lower in those with CSBD compared to both the RSB and the C groups. In studies involving clinical groups, few results refer to the past-positive TP. This is understandable, due to its specific function, which is expressed in positive associations between the severity of this trait and health, well-being, self-esteem, happiness, life satisfaction or health behavior [39,40,41,42,44,45,46]. The protective effect of this TP in the development of mental disorders has been shown, for example, in relation to the severity of depressive and anxiety symptoms [41,82,104]. Therefore, it is worth interpreting our results of people with CSBD in opposition to research on protective factors in health maintenance. One can then infer the lack of a protective effect of this perspective on mental health, and deficits in its buffering effect against past-negative TP. Similar results were noted in a study by Unger et al. [62], in reference to the role of low past-positive TP in the severity of compulsive buying symptoms, but only in one of the three groups studied: the students from China. No such difference was shown in students from Ukraine and Germany. Reduced past-positive TP also characterized the group with a diagnosis of gambling disorder, compared to social gamblers, in a study by Hodgins and Engel [66].

### 4.3. Present-Hedonistic

In view of the results of previous studies looking at the severity of behavioral addiction symptoms, including compulsive buying [62], Internet, social media use [68,91,92] and gambling disorders [64,94], the lack of differences in the severity of present and future hedonic perspective between men with CSBD and RSB and the C group may seem unexpected. Our own study did not predict such differences, but both the reasons for this assumption and the results of the study warrant comment.

Similar, though more surprising, results were obtained in the previously mentioned study of Polish FB users, in which present-hedonistic TP was found to be a negative predictor of Internet use disorder [67]. As indicated by other researchers [105,106,107] and the responses provided by the participants in this study, the use of pornographic websites is often an important form of realization of sexual behaviors associated with CSBD. Perhaps both Internet use disorder and CSBD are different from substance use or gambling disorder [67,108] and have a common denominator that has not yet been identified.

Based on diagnostic criteria [21,100], studies and concepts indicating a compulsive or typical mechanism for addiction in CSBD [24,35,109,110,111], it might be suspected that enjoyment is not the main reason for engaging in sexual activities. Rather, it should be expected that motives relate to the regulation of unpleasant, overly strong emotions by sexual activities or the build-up of sexual arousal in response to a negative mood [112,113]. Motives related to the inability to inhibit a reaction in response to situational cues and even a decrease in pleasure and the need to intensify sexual behavior are also considered, analogous to the mechanisms of substance dependence [24,105,109,114]. 

A different type of inclusion into groups and other related statistical analyses are also considered hypothetical reasons for the lack of differences in the severity of the present hedonistic TP in men in the CSBD and C group. In many studies, individuals for whom characteristics typical of disorders due to substance use or addictive behaviors are used are qualified or classified based on the results of screening tests or other tests estimating the severity of the problem [62,67,68]. Sometimes the division into a group with clinical symptoms and control is not made at all, but only refers to the severity of the trait, tendency or behavior of interest to the researchers, such as in studies on compulsive buying [62]. In the case of our own research, men with CSBD were clearly separated, not only from the C group (without CSBD symptoms and RSB), but also from the RSB group (without CSBD symptoms). In this way, relatively homogenous study groups were obtained. This showed that a generalized focus on providing strong, positive experiences quickly without considering their potential consequences, but also simply enjoying day-to-day experiences, is not typical of men with CSBD, but is a distinguishing feature of those with RSB. In addition to the aforementioned, positive aspects of this TP include the ability to find, take pleasure in and enjoy current moments, activities or interpersonal contacts. A threat may be the poor ability to defer gratification, sometimes even in the face of potential harm [39,40,41,42]. Thus, the result obtained is in line with TPT assumptions [39,40], as well as studies confirming the links between the present-hedonistic and risky behavior, i.e., risky driving [48,50], alcohol drinking and drug use [43,48,56] or smoking [48,56].

Available studies involving individuals with RSB that can be directly referenced (ZTPI was used) are those by Henson et al. [48], Sosy-Rubi et al. [115] and a preliminary report by Sullivan and Barkley-Levenson [116]. The former looks at the associations of TP with a range of pro- and anti-health behaviors, which included RSB. The results of 1568 college students (age M = 19.3 years) confirmed the significance of a present-hedonistic present TP as a predictor of the number of sexual partners in the past year and throughout the person’s lifetime [48]. In contrast, a study involving 65 young adults (age M = 22.72 years) did not confirm the important role of the present-hedonistic TP in RSB [116]. Additionally, a study of 326 men who have sex with men, whose median age was 23, shows that the present orientation reduces the likelihood of condom use with regular, but not with casual partners [115]. In general, the results presented above are similar to those obtained in the presented study, but they do not refer to the overall rate of RSB but to specific individual behaviors, mainly increasing the probability of STIs, and the people included in the studies are younger and more specific in other characteristics. Therefore, the result obtained suggests that the present-hedonistic TP may be a factor that increases the chances of undertaking a variety of RSB, regardless of the specificity of the population, and it is worth extending the research in this regard.

### 4.4. Present-Fatalistic and Future

As with the present-hedonistic TP, the lack of differences in the severity of the future TP and the lower level of the present-fatalistic TP in men with CSBD compared to the C group is not consistent with at least some of the previous observations, such as for symptoms of gambling disorder [66,95], Internet or social media use disorders [67,68,70,91,92] and compulsive buying [62]. On the other hand, this is not surprising and is not an isolated result [61,62,71,73].

In our study, no differences were expected in this regard, due to the characteristics of the clinical group. It was supposed that those who choose to enter therapy will not have a strong tendency to allocate responsibility for their own current situation to unidentified “forces,” lack of luck or solely the actions of other people. In addition, patients who persist in therapy will be inclined to set distant, even difficult goals and think hopefully about the possibility of achieving them. This is confirmed by studies in which the future perspective allowed for predicting positive outcomes of alcohol dependence treatment [60,117]. Therefore, the results on the future and present-fatalistic scales do not seem specific to CSBD, but connect them more with people deciding to make changes in their lives, to take care of themselves, such as by entering treatment. In this sense, a possible limitation of our application to patients with CSBD who attend therapy is suggested, with the indication that the use of therapy may be an important feature for the results obtained. Therefore, it seems important to undertake comparative studies of people with CSBD and/or other behavioral addictions who are only given this diagnosis with those who additionally benefit from therapy.

The CSBD group result is similar to other groups’ abilities to set distant goals and strive to achieve them, demonstrating optimism and belief in the meaningfulness of efforts sustaining result-oriented activity. Men with CSBD have a real, even weaker tendency to attribute their current situation to fate or other external forces. These traits appear to share characteristics with psychological constructs such as an internal locus of control [118], task-oriented stress coping style [119], dispositional optimism [120], self-efficacy [121] and sense of coherence [122]. Our results may also be supported by research showing negative relationships between present-fatalistic and active coping styles, positive self-esteem, or dispositional optimism [104], positive relationships of future intensity with active coping strategies [58,104], impulse control [39] and internal locus of control [123]. Additionally, both low levels of present-fatalistic and even higher levels of future TP are associated with achieving professional and academic success [124], life satisfaction [45,78] and engaging in health behaviors [46,48,125]. Therefore, both findings can be interpreted as a resource that can probably be used effectively in therapy at many points.

### 4.5. Balanced Time Perspective

Analyses showed that the TP profile of those with CSBD deviated more from the BTP than that of men in the C group. This result is in line with previous findings indicating that DBPT can be an indicator of the level of health. DBTP is associated with poorer mental and physical functioning, higher levels of anxiety, depressiveness or poorer performance [54,55,126], while the greater balance of TP correlates with and is a predictor of well-being, self-actualization, positive mood, good performance and job satisfaction and quality of interpersonal relationships [45,52,53,54,75,126]. With regard to behavioral addictions, DBTP has rarely been considered to date. For example, it has been proven that individuals with a more balanced TP were characterized by a lower propensity for compulsive buying [62] and food addiction [74]. Unfortunately, on the basis of the DBTP value alone, it is not possible to infer the mental ability to effectively switch between TP depending on situational demands, tasks and goals. This is precisely how the adaptive function of a BTP was originally understood [39,40]. However, to date, the flexible switching and use of particular TP has not been operationalized, so this aspect is difficult to identify in research. Regardless of the aforementioned shortcomings, the result obtained can be regarded as reinforcing DBTP significance, the usefulness and operationalization of which are still being studied and discussed [54,55,127].

For men with CSBD, relationships between past TP are common, shared with those identified in other addictions and psychiatric disorders more broadly. Over-focusing and interpreting current events through a past-negative lens poses a health risk, while scant recall and reliance on positive memories deprive these individuals of a buffering factor for mental health protection. Present-hedonistic as a distinguishing feature of men with RSB is typical of risk-taking behavior in general, but rarely reveals itself as the sole distinguishing perspective. Arguably, the RSB group we studied is unique compared to other studies of risky behavior. It includes men who are generally quite healthy, without CSBD features. Therefore, the result is more indicative of a protective profile in the development of addiction than a risk for its development. This can be tested in further studies considering groups of people with high RSB with different severity of CSBD symptoms. TP-based interventions, optimizing TP profiles toward BTP are worth considering in future experimental studies and evaluations. Future studies need to include women. They pursue therapy less frequently, making it more difficult to reach this group. Nevertheless, the problem of CSBD is present and poorly understood in this population. Individual data suggest that the problematic nature of both the symptoms themselves and the psychological features associated with CSBD in women may be more specific, and separate from men’s [33,128,129].

### 4.6. Practical Implications

The results of our study are largely in line with the predictions of the TPT and previous research on similar topics. Therefore, therapeutic programs can be developed based on these predictions. An example of a therapy based on TPT is Time Perspective Therapy. It is based on defining the profile of time perspectives and working on a better balance, mainly promoting a more positive orientation to the future instead of a negative past. Ultimately, it will help achieve better responsiveness and health [130,131]. Time Perspective Therapy has so far been used not only to treat patients with PTSD [130,131], but also those with substance use disorders [72], with promising results. Another form of therapy related to TPT, Future Oriented Group Training, effectively reduces stress levels, depressive symptoms and suicidal thoughts in individuals with suicidal tendencies [132].

When considering the possible use of therapy based on TPT for persons with CSBD, it seems most important to consider the relationship between the lower intensity of the past-positive (pro-health) perspective and the higher intensity of the past-negative (anti-health) perspective. Another perspective that can be developed is present-hedonistic, but in its pro-health aspects. In the work on constructively strengthening this perspective, there is an intentional focus on current sensations, pleasures and even their deliberate planning and treating them as important in everyday activities. This is achieved with the use of physical activity or methods such as mindfulness, yoga, meditation. These methods help people to become aware of sensations and thoughts without the habit of evaluating them and automatically responding to every experienced need. These interventions also improve effective emotional self-regulation and self-control [133]. Mindfulness-based relapse prevention is useful in substance dependence treatment [134] and, as the first pilot study indicated, could be also beneficial for CSBD individuals [135]. The resources of men with CSBD seem to include a typical or even higher level of self-agency and goal orientation. This is a resource that can be relied on in the course of therapy [60,117].

It also seems essential to design preventive measures for people who engage in risky sexual behavior. However, it is necessary to consider this group’s time perspective profile characteristic, namely the tendency towards present-hedonistic TP. Prevention programs could refer to the future as standard but consider the needs realized through behaviors related to satisfaction, pleasure and immediate gratification. It sometimes exceeds the health or moral recommendations preferred in a given community. In this case, the goal would be not to prevent the occurrence of specific behaviors but to minimize the risk and negative consequences. It seems that the National AIDS Center [136] adopts a similar approach in Poland, establishing Consultation and Diagnostic Points, where not only HIV testing is carried out but also individual anonymous education and counseling.

### 4.7. Limitations

The study has several important limitations. The first is related to the properties of the tool itself. The ZTPI takes into account all three time areas, but the future scale does not distinguish its positive and negative aspects, which causes interpretive uncertainty. Consequently, it can lead to treating the future only in terms of goal orientation and optimism. This issue has already been raised [42], leading to the separation of subscales analogous to past, future-positive and negative TP. It seems that such an approach can add much more to the understanding of the importance of the future perspective, especially in clinical groups, in which negative thoughts are probably not limited to the ruminations of past traumas and failures, but enter the future. Such a distinction was used, for example, in the study previously mentioned by Stolarski et al. [81]. Consequently, we suspect that our conclusions regarding future perspective may also be characterized by some imperfection and interpretive uncertainty.

Data collection relied on the results of questionnaire surveys, and while psychometric characteristics can be relied on for the most relevant traits, clinical and demographic data collection did not use objective data (including medical records, laboratory test results, etc.), but relied only on participants’ declarations. 

All groups were dominated by men who did not confirm health problems. The number of diagnoses of somatic diseases and behavioral addictions (except for CSBD) does not differentiate the studied groups. However, the prevalence of mental disorders and substance abuse is higher in the CSBD group. This distribution was in agreement with predictions and represents some important characteristics of CSBD patients, which should be taken into account in research and treatment [25,137]. Excluding men with multimorbidity from the study would remove potentially confounding variables, but would result in conclusions that have little to do with clinical realities. However, this limitation is worth bearing in mind when considering the study’s conclusions and planning future selecting to clinical and control or comparative groups.

## 5. Conclusions

The intensity and relationships between the TP domains of patients with CSBD indicate a stronger tendency to focus on negative rather than positive experiences from the past compared to non-CSBD men, both engaging in and not engaging in RSB. The time perspective profile of RSB men is similar to those of men who do not exhibit RSB. The distinguishing feature of men with RSB without CSBD is a greater ability to enjoy current experiences. Using the profile of temporal characteristics of a men engaged in RSB and with CSBD may increase the effectiveness of prevention programs and therapeutic interventions.

## Figures and Tables

**Table 1:** Specific inclusion and exclusion criteria for each group in the study.

Group	Inclusion Criteria	Exclusion Criteria
Men with Compulsive Sexual Behavior Disorder (CSBD, clinical group)	- CSBD diagnosis by a specialist - Current treatment or specialist help-seeking (diagnosis, therapy) in connection with CSBD	
Men undertaking Risky Sexual Behavior (RSB, comparative group)	- High intensity of RSB (RSB scale scores from 5 to 10 points)	- CSBD diagnosis by a specialist - A positive result of the screening sexual addiction assessment (SAST-R scores from 5 to 20 points)
Men without CSBD and not engaged in RSB—Control group (C)	- Low or moderate intensity of RSB (RSB scale scores from 0 to 4 points)	- CSBD diagnosis by a specialist - A positive result of the screening sexual addiction assessment (SAST-R scores from 5 to 20 points)

CSBD—Compulsive Sexual Behavior Disorder, RSB—Risky Sexual Behavior, SAST-R—The Sexual Addiction Screening Test-Revised, C—Control group.

**Table 2:** The level of reliability of the tools used in the study.

		95% CI	
	Cronbach’s Alpha	LL	UL
Zimbardo Time Perspective Inventory			
Past-negative	0.83	0.81	0.85
Present-hedonistic	0.77	0.74	0.80
Future	0.76	0.73	0.79
Past-positive	0.71	0.67	0.74
Present-fatalistic	0.73	0.69	0.76
The Sexual Addiction Screening Test-Revised	0.90	0.89	0.92
Risky Sexual Behavior scale	0.76	0.72	0.79

CI—confidence interval, LL—lower limit, UL—upper limit.

**Table 3:** Demographic characteristics of studied groups.

	Compulsive Sexual Behavior Disorder Group (N = 98)		Risky Sexual Behavior Group (N = 63)		Control Group (N = 264)	
	N	%	N	%	N	%
Education						
Primary	1	1	0	0	0	0
Vocational	6	6.1	4	6.3	36	13.6
Secondary	39	39.8	25	39.7	95	36
Higher	52	53.1	34	54	133	50.4
Employment						
Currently working	93	94.9	58	92.1	231	87.5
Student	2	2	5	7.9	21	8
Unemployment	3	3.1	0	0	12	4.5
Partner Status						
In steady relationship	64	65.3	41	65.1	226	85.6
No steady relationship	34	34.7	22	34.9	38	16.4
Psychosexual Orientation						
Heterosexual	85	86.7	58	92.1	261	98.9
Homosexual	6	6.1	4	6.3	1	0.4
Bisexual	6	6.1	0	0	2	0.8
Asexual	1	1	1	1.6	0	0
Gender of Previous Sexual Partners						
Female	77	78.6	57	90.5	230	87.1
Male	5	5.1	5	7.9	4	1.5
Female and male	9	9.2	0	0	2	0.8
Lack of sexual relations	7	7.1	1	1.6	28	10.6

**Table 4:** Clinical characteristics of studied groups.

	Compulsive Sexual Behavior Disorder Group (N = 98)		Risky Sexual Behaviors Group (N = 63)		Control Group (N = 264)	
	N	%	N	%	N	%
Substance Use Disorders						
No	73	74.5	51	80.9	242	91.7
Single substance dependence	20	20.4	9	14.3	20	7.6
Dual or more substances dependence	5	5.1	3	4.7	2	0.8
Alcohol	21	21.4	4	6.3	5	1.9
Smoking (nicotine)	8	8.2	9	14.3	15	5.7
Marijuana	5	5.1	2	3.2	2	0.8
Amphetamine	3	3.1	1	1.6	0	0
Opiates	3	3.1	0	0	0	0
Cocaine	1	1	0	0	0	0
Medications	2	2	1	1.6	0	0
Addictive Behaviors						
No	93	94.9	58	92.1	255	96.6
Single addictive behavior	4	4.1	3	4.7	6	2.3
Dual or more addictive behaviors	1	1	2	3.2	3	1.1
Gambling	1	1	2	3.2	1	0.4
Internet	5	5.1	3	4.7	6	2.3
Food	1	1	2	3.2	2	0.8
Exercises	2	2	1	1.6	2	0.8
Buying	1	1	0	0	0	0
Mental Disorders						
No	75	76.5	59	93.6	252	95.5
One disorder	19	19.4	4	6.3	10	3.8
Two or more disorders	4	4.1	1	1.6	2	0.8
Depressive disorders	16	16.3	2	3.2	4	1.5
Anxiety disorders	4	4.1	2	3.2	6	2.3
Personality disorders	3	3.1	0	0	3	1.1
Bipolar affective disorder	2	2	1	1.6	1	0.4
Other mental disorders	2	2	0	0	0	0
Chronic Somatic Diseases						
No	80	81.6	58	92.1	229	86.7
One disease	16	16.3	4	6.3	35	13.3
Two or more diseases	2	2	1	1.6	0	0
Hypertension	6	6.1	4	6.3	24	9.1
Diabetes	1	1	0	0	5	1.9
Asthma	2	2	0	0	2	0.8
Irritable bowel syndrome	3	3.1	0	0	0	0
Hypothyroidism	1	1	0	0	1	0.4
Cardiac arrhythmias	0	0	1	1.6	1	0.4
Other diseases	5	5.1	1	1.6	2	0.8

**Table 5:** Level of time perspectives and deviation from balanced time perspective in the studied groups.

	Compulsive Sexual Behavior Disorder Group (N = 98)		Risky Sexual Behavior Group (N = 63)		Control Group (N = 264)		*H*	*p*
	*M*	*SD*	*M*	*SD*	*M*	*SD*		
Past-negative	3.01	0.72	2.66	0.83	2.80	0.74	9.81	0.007
Present-hedonistic	3.18	0.54	3.45	0.56	3.22	0.55	9.24	0.010
Future	3.43	0.54	3.34	0.60	3.49	0.58	3.39	0.184
Past-positive	3.21	0.67	3.58	0.56	3.57	0.64	19.43	<0.001
Present-fatalistic	2.43	0.81	2.69	0.73	2.63	0.65	7.41	0.025
Deviation from the Balanced Time Perspective	2.53	0.76	2.29	0.75	2.30	0.72	7.84	0.020

## Data Availability

The data presented in this study are available on request from the corresponding author.
